# The Influence of Selected Test Conditions on the Impact Strength of Adhesively Bonded Connections

**DOI:** 10.3390/ma13061320

**Published:** 2020-03-14

**Authors:** Jan Godzimirski, Andrzej Komorek

**Affiliations:** 1 Department of Mechatronics and Aviation, Military University of Technology, 00-908 Warszawa, Poland; 2 Department of Aviation, Polish Air Force University, 08-530 Deblin, Poland

**Keywords:** adhesive connections, impact strength, numerical calculations

## Abstract

The conditions of adhesive connections testing can significantly affect the recorded findings. The standards, under which the investigations are conducted, do not take into account numerous factors that can greatly influence the outcome of the tests. Moreover, the research parameters in the standards are not specified. It is not defined in what manner their change, or any failure to comply with the standards, will affect test results. This article presents the results of experimental research, completed with numerical simulations, designed to test to what extent the recorded impact strength of adhesive connections is affected by the stiffness of the test stand, and the speed and energy of the impactor. In the experimental research, the authors used block samples whose substrates were made of an aluminum alloy. The elements of the samples were bonded by means of three different epoxy adhesives. The models used in numerical calculations were built on the basis of the real dimensions of the specimens used in this experimental research. As a result of the performed tests and conducted calculations, it was found that the use of test stands of lesser stiffness resulted in an increased registered impact strength due to an increased energy value of elastic deformations.

## 1. Introduction

Examinations of impact strength of adhesive connections are becoming increasingly popular and necessary due to the constantly rising overall use of this type of connection in contemporary design, and due to the fact that glued structures may undergo an impact load. 

Dynamic, single, or multiple loads often occur during the operation of means of transport [1,2,3,4] or when using mobile phones [5]. Designers, in their assumptions, do not frequently take into account the effects of impact loading, or only estimate these impacts with a great deal of approximation. This is quite understandable in numerous cases, as it is difficult to enter into the design numerical values of the impact strength of the materials or connections that will be used. However, it seems justified to change this situation by seeking a universal method that allows for the determination of impact strength, whose value might be applied in construction calculations. The currently used methods of impact strength testing of adhesive connections produce results that can be used solely for the comparative assessment of exploited glues in specific types of connections [6,7]. Other applications of the results prove to be impossible in most cases. Difficulties of interpretation also arise from the same research methodologies applied with differences in the conditions of research and sample preparation [8,9,10]. In addition, the stiffness of the test stand appears to be of key importance to the reliability of the results, as it is usually disregarded.

One of the most popular methods of impact strength testing of glued connections is the standard method [11,12,13]. This research technique includes many of the described features, therefore it was decided to carry out investigations that will make it possible to assess the influence of certain factors on the obtained results. The article is intended to check to what extent the measured impact strength of adhesive connections is affected by the stiffness of the test stand, and the load speed and energy of the impactor.

## 2. Preparation of Samples

**Figure 1:**
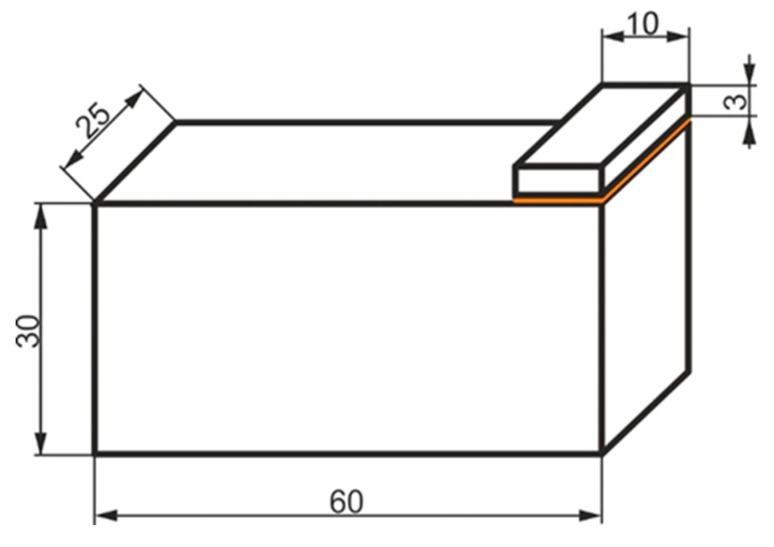
Sample used in impact strength tests. All dimensions in (mm).

In order to test the impact strength of the samples, the authors prepared batches of adhesively bonded block samples (Figure 1), whose adherends were made from aluminum alloy 2017A.

The adhesive materials were Epidian 57 with Z1 hardener, as well as construction adhesives Loctite 3421 and 3430, selected due to their significant difference in the Young’s modulus (for Epidian 57/Z1−E = 1800 MPa, for Loctite 3421−E = 965 MPa, and for Loctite 3430−E = 3210 MPa). The thicknesses of the joints were the same and measured 0.1 mm.

Prior to bonding, the metal parts were cleaned and their surface was given proper structure and roughness through abrasive blasting [14,15,16], with copper slag as an abrasive medium. Then, the surfaces of the samples were washed with petroleum ether in order to remove the remaining dust after the abrasive blasting treatment and the grease from the samples. In order to evaporate the petroleum from the surface of the samples, the washed samples were placed in a drying chamber SLW 53 (POL-EKO, Wodzisław Śląski, Poland) at 90 °C. The prepared substrates were glued as soon as possible to avoid accidental soiling of the bonded surfaces or the falling of dust, oxides, or moisture. While fixing the items for bonding, particular attention was paid to proper positioning of the elements against each other, as even slight irregularities in the geometry of the samples may lead to significant changes in the obtained findings. The bonded series of samples with identical joint thickness were placed on a base plate and clamped down at a pressure of 40 kPa for the time of curing, which equaled 7 days at ambient temperature (21 °C).

After curing the joints, the authors performed a visual inspection of the quality of the obtained connections and removed excess glue and threads outside the bonded surfaces. Removing the threads soaked in glue was particularly relevant, since they may be caught up during an impact strength test by a torn upper element and consequently slow down the dropping tool, which would translate into a higher value of impact strength of the connection. The excess glue may result in raising the strength of the joint [17] (in practice, they are not removed unless it is necessary).

In order to carry out the investigations, the authors prepared 132 samples, which were further divided into 3 groups. Each group consisted of 44 samples, which were joined with a different glue. In addition, each of the 3 groups was divided into 4 sets of 11 samples that were tested under identical conditions. 

## 3. Experimental Research

The examination was carried out on a specialized pendulum hammer Julietta (Institute for Sustainable Technologies-National Research Institute, Radom, Poland), in which it is possible to use two pendulums of different maximum energy of 10 J or 25 J. The pendulum of 10 J has a smaller flexural stiffness (arm diameter of 12 mm), whereas the pendulum of 25 J has higher bending stiffness (arm diameter of 20 mm). Moreover, the testing device allows for the modification of the initial energy of the impactor through moving the angle of the pendulum, which is also associated with changing the speed of the impact. The maximum shifting of the pendulum loads the examined sample at a speed of 3.81 m/s. 

In order to check whether the eleven-piece batch of samples is sufficiently large to conduct impact strength tests, for all the examined adhesives the authors conducted testing of two eleven-piece batches in the same conditions and then compared their results (Table 1). The investigations were performed by applying the pendulum of 10 J maximum energy and an impact speed of 3.81 m/s.

**Figure 2:**
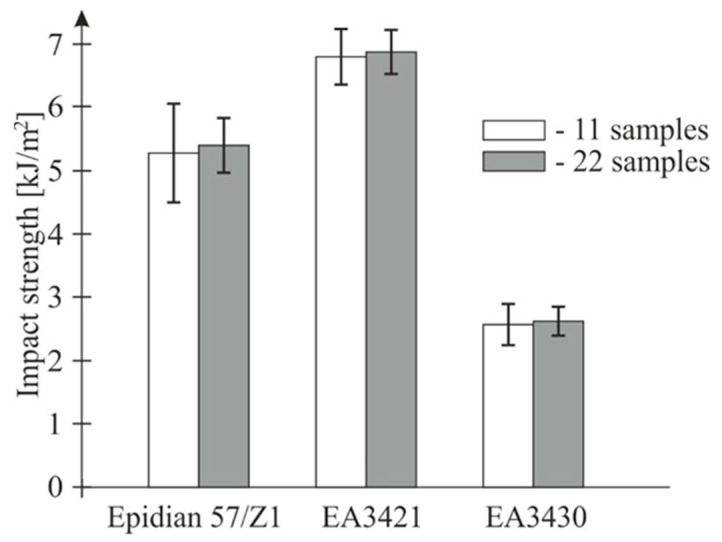
Average impact strength of the three adhesives and a standard deviation of the eleven-piece and twenty-two-piece batch (10 J hammer, speed of 3.81 m/s).

In Figure 2, there is a comparison of an eleven-piece batch with a twenty-two-piece batch. As expected, there was little difference in the mean value and a lesser standard deviation of a larger sample batch. It was assumed that further investigations could be performed on eleven-piece sample batches.

**Figure 3:**
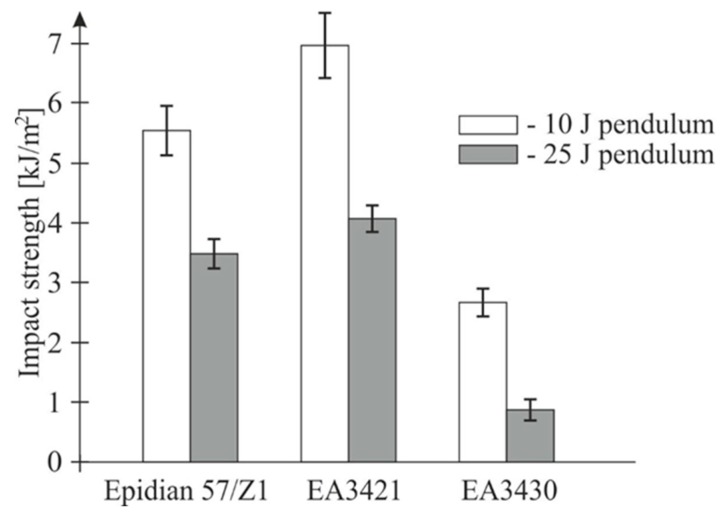
Impact strength of three adhesives for V = 3.81 m/s, using the pendulums of 10 J and 25 J.

In order to evaluate the influence of the pendulum stiffness upon the impact strength of block connections, batches of identical specimens were loaded by a pendulum of 10 J and 25 J, at a speed of 3.81 m/s. The findings of the tests are presented in Figure 3. 

The registered lesser impact strength of samples loaded with the pendulum of 25 J may be explained by a smaller value of the deformation energy of the stiffer pendulum arm “25 J” (Φ = 20 mm) than of the “10 J” (Φ = 12 mm). This might indicate a considerable impact of the test stand stiffness upon the recorded findings. 

**Figure 4:**
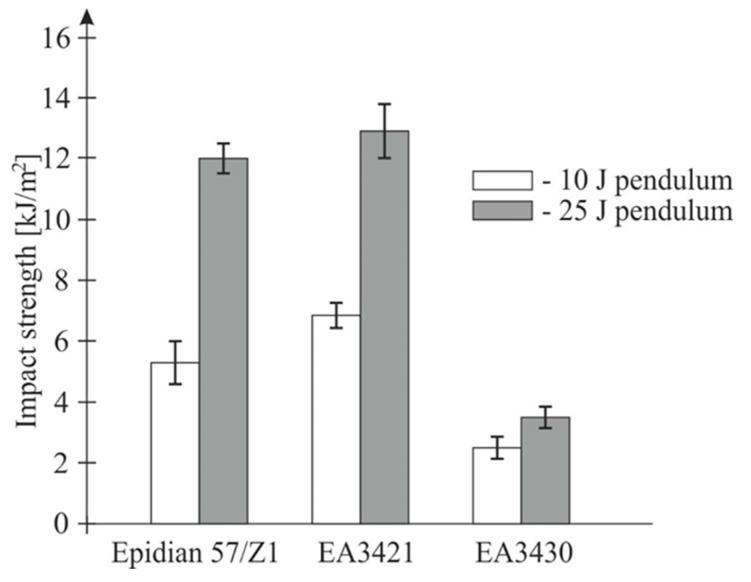
The impact strength of the three adhesives for the test energy Ek = 10 J, by means of pendulums “10 J” (V = 3.81 m/s) and the “25 J” (V = 2.42 m/s).

Additionally, investigations were conducted in which identical samples were loaded with the energy of 10 J using different pendulums (“10 J” and “J 25”). The pendulum “10 J” struck the sample at a speed of 3.81 m/s, whereas the pendulum “25 J” struck at a speed of 2.42 m/s. The findings of these tests are presented in Figure 4.

The obtained results suggest that reducing the impact speed increases the recorded impact strength of adhesive connections. However, taking into consideration greater stiffness of the pendulum “25 J”, the result requires a more in-depth analysis.

## 4. Numerical Calculations

**Figure 5:**
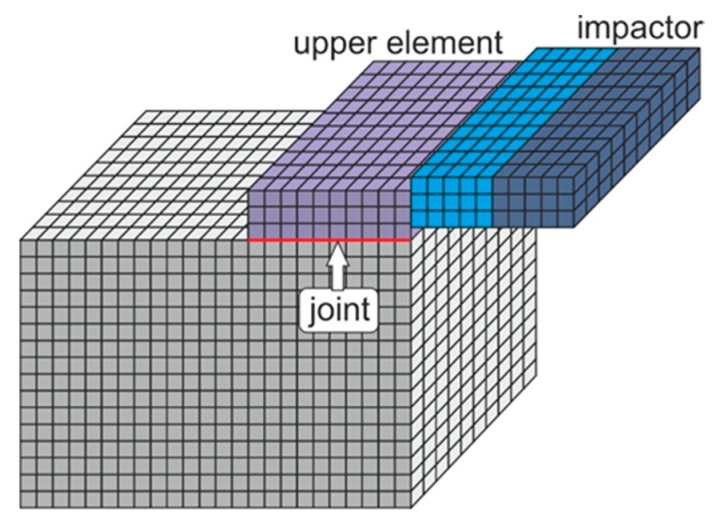
Model of the block sample, dynamically loaded.

The calculations were conducted in the software ANSYS 16.2, using the Explicit Dynamics module. A numerical model of the tested samples was built (Figure 5).

**Figure 6:**
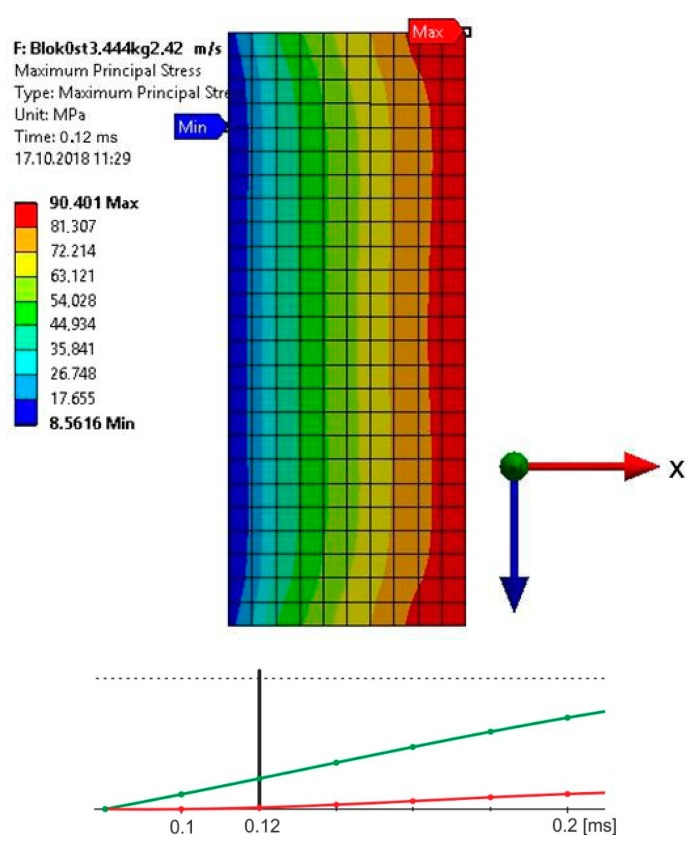
Maximum principal stress distribution for the computing time of 0.12 ms in the joint, loaded with energy 10 J, at a speed of 2.42 m/s.

**Figure 7:**
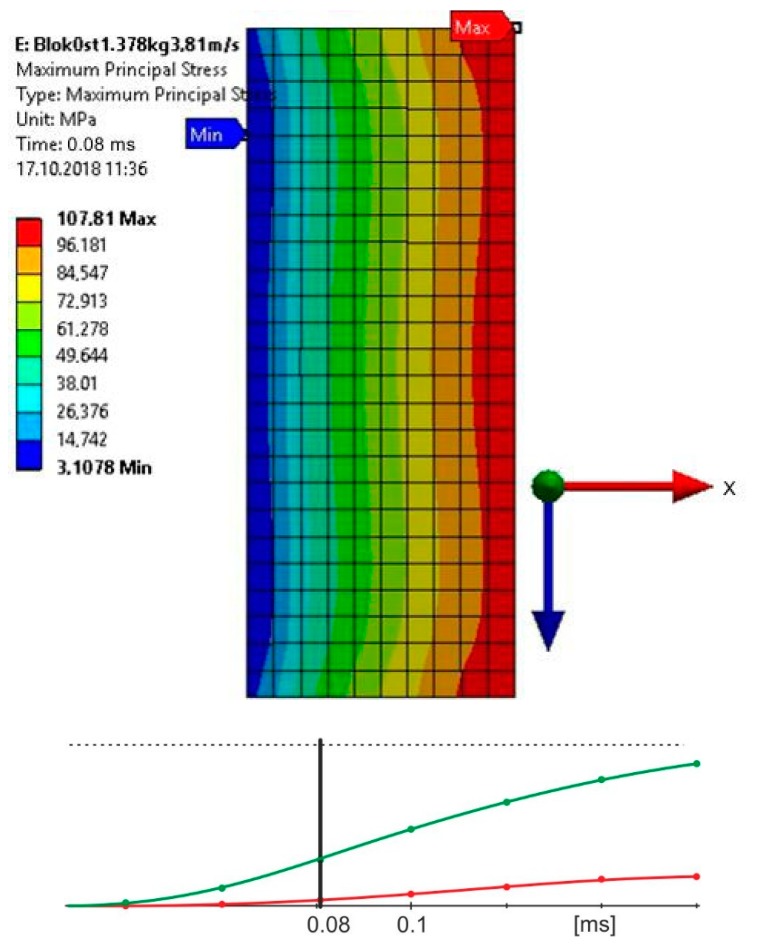
Maximum principal stress distribution for the computing time of 0.08 ms, in the joint loaded with the energy of 10 J, at a speed of 3.81 m/s.

The impactor was modelled with two elements, in which the striking piece that directly struck the upper element of the sample had a stiffness equal to steel (E = 200 GPa); the stiffness of the other element was higher by one row in value (E = 2000 GPa) in order to ensure adequate stiffness of the impactor [18]. The material of the impactor was given appropriate density to ensure that, during the impact speeds of 3.81 and 2.42 m/s, the impact energy equaled 10 J. Taking into account the fact that in the course of the experimental research there was no plastic deformation of the upper element, the calculations were carried out for linear-elastic deformations of the examined sample. The assumed properties of the adhesive joint (E = 2 GPa, ν = 0.35) corresponded to the properties of the adhesive Epidian 57/Z1. The authors assumed the computing time of 0.4 ms. The obtained stress distribution in the joint for certain times relied on the speed of the load. The authors estimated the time, after which, in the joints, the maximum principal stresses were close to the destructive stresses of the adhesive Epidian 57/Z1 [19] (Figure 6 and Figure 7).

**Figure 8:**
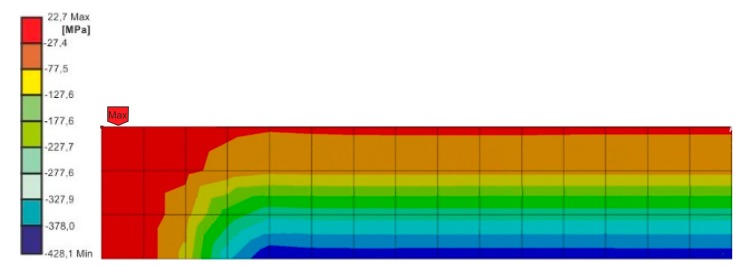

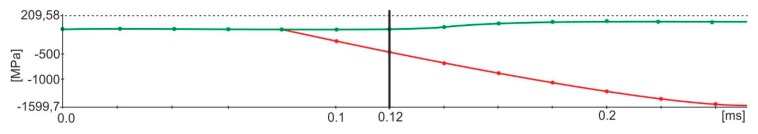
Normal stress distribution on the half of the frontal surface of the overlay, for a computation time of 0.12 ms, loaded with energy 10 J, at a speed of 2.42 m/s.

**Figure 9:**
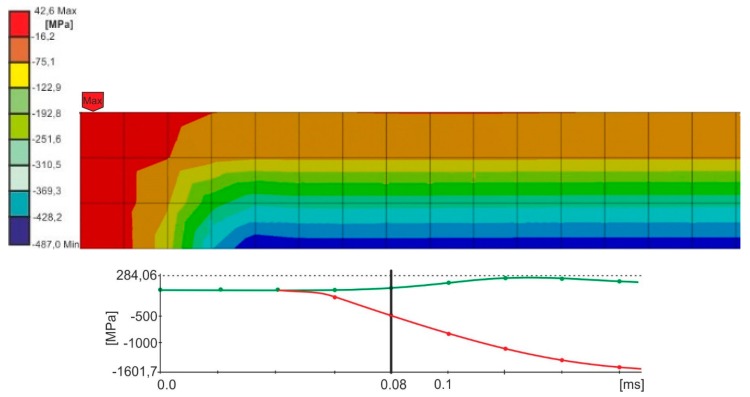
Normal stress distribution on the half of the frontal surface of the overlay, for a computation time of 0.08 ms, loaded with energy 10 J, at a speed of 3.81 m/s.

Based on the conducted calculations, it appears that at the load speed of 2.42 m/s, the stresses that were close to the destructive ones in the joint occurred at the computation time of 1.2 × 10^−4^, and at the speed of 3.81 m/s and the time of 8 × 10^−5^. For these times, it was possible to estimate the force of the impactor striking the spacer during the time of the occurrence of the stresses in the joint, being close to the destructive ones, based on stresses σx (Figure 8 and Figure 9).

**Figure 10:**
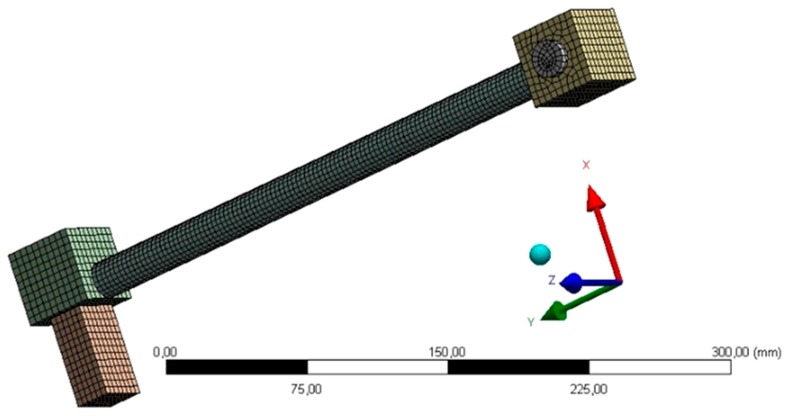
Numerical model of the pendulum Φ = 20 mm.

The conducted analysis demonstrates that for both loads under scrutiny, the force had a similar value of about 8000 N. It can be assumed that the greater impact strength of the samples, loaded at a lower speed, resulted from an increased elastic deformation energy of the more slowly loaded pendulum. The authors modelled a pendulum Φ = 20 mm (Figure 10) and loaded it with an energy of 3 J, first at a speed of 3.81 m/s and then at a speed of 2.42 m/s. The energy of 3 J was obtained in both cases, giving the striking element a suitable density.

**Figure 11:**
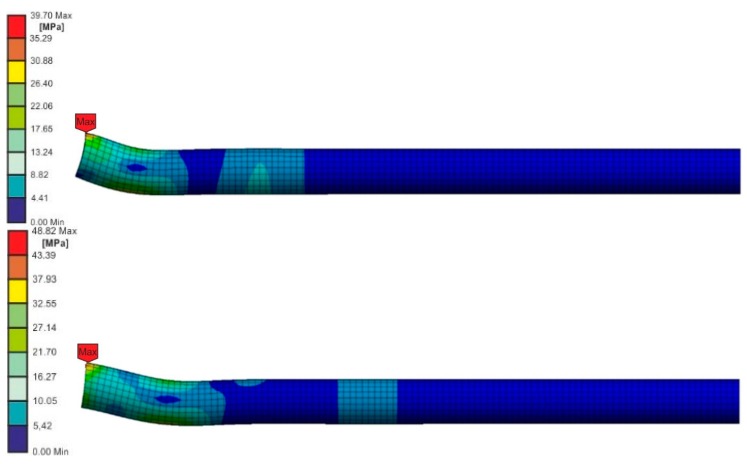
Von Mises stress distribution in the pendulum arm, loaded with the energy of 3 J at the speeds 3.81 m/s (upper picture) and 2.42 m/s (bottom picture) at stress in joint close to the adhesive strength.

The authors compared von Mises stresses in the pendulum arm loaded at the speeds of 3.81 m/s and 2.42 m/s for the computation times, for which there were stresses close to the resistance of the adhesives (Figure 11).

On the basis of the numerical calculations, it appears that at the time of the destruction of the joint in the pendulum arm loaded with a higher speed, there were lesser stresses in the smaller material volume, and thus the elastic energy of the deformations was smaller than in the case of the load at a slower speed. It seems that the pendulum that is impact-loaded more slowly absorbs more energy of elastic deformations. The recorded energy is a sum of the energy of the joint’s destruction and elastic deformations of the test stand.

## 5. Conclusions

(1) The recorded energy of impact damage of adhesively bonded connections is a sum of the energy of the joint’s destruction and the elastic deformations of the test stand.
(2) The use of research stands of lesser stiffness resulted in increasing the registered impact strength due to a higher energy value of elastic deformations.
(3) Reducing the speed of impact loading in research investigations led to an extended time of the research stand, and thus an increase in the elastic strain energy. Therefore, it caused an increase in the recorded impact strength.
(4) The comparative study of the impact strength of adhesive connections should be executed on test devices, whose stiffness and speed of load application has been explicitly specified.

## Figures and Tables

**Table 1:** Results of impact strength testing of samples bonded with various adhesives.

Adhesive	Loading Conditions	Type of Pendulum	Mean Impact Strength [kJ/m^2^]	Standard Deviation [kJ/m^2^]	Confidence Interval [kJ/m^2^]
Epidian 57/Z1	Ek = 10 J v = 3.81 m/s	10 J	5.29	±0.78	±1.74
			5.54	±0.4	±0.89
EA 3421	Ek = 10 J v = 3.81 m/s	10 J	6.86	±0.44	±0.98
			6.93	±0.56	±1.25
EA 3430	Ek = 10 J v = 3.81 m/s	10 J	2.56	±0.34	±0.76
			2.69	±0.25	±0.56
